# Shared Developmental Trajectories for Fractional Reasoning and Fine Motor Ability in 4 and 5 Year Olds

**DOI:** 10.3390/bs11020026

**Published:** 2021-02-15

**Authors:** Lindsey Clark, John Shelley-Tremblay, Julie Cwikla

**Affiliations:** 1 Department of Psychology, University of South Alabama, Mobile, AL 36688, USA; leclark6@crimson.ua.edu; 2 Creativity & Innovation in STEM, University of Southern Mississippi, Hattiesburg, MS 39406, USA; julie.cwikla@usm.edu

**Keywords:** numerical cognition, fractional reasoning, fine motor ability, gesture

## Abstract

We investigated preschool-aged children’s understanding of early fractional tasks and how that performance correlates with fine motor skills and use of gestures while counting. Participants were 33 preschoolers aged 4 to 5 in two Southeastern public elementary schools. Children were tested individually in an interview-like setting. Mathematics tasks were presented in a paper and pencil format and the Grooved Pegboard test assessed fine motor skills. Finally, utilization of gestures was evaluated by taking a behavioral rating of the child’s hand morphology, accuracy of gestures, and synchrony of gestures and spoken word while performing a counting task. Results indicate that performance on fractional reasoning tasks significantly predicts both fine motor ability and accuracy of gestures.

## 1. Introduction

Few studies have examined the ability of children between the ages of three and five years old to solve fraction problems without formal instruction [1]. Starting from birth, humans have the capacity to mentally discriminate between different numerosities, an ability known as our number sense [2]. During development, number knowledge gives us an understanding of the cardinal principle, meaning that the final word in a count sequence represents the amount of the entire set. This understanding has been linked to more advanced ideas such as fairness and equivalence and some researchers believe these judgments could be a longitudinal predictor of how well a child performs in arithmetic [3]. Thus, numerical knowledge is a critical cognitive milestone as it allows children to understand notions of fairness and equal sharing between one or more parties [3]. In addition, cognitive development around this age also displays a corresponding protracted time course with fine motor development [4]. Supporting research [4,5] proposes that these two facets of development are in some way intertwined. Furthermore, recent research supports an active sensorimotor system presence when working with numbers, suggesting a link between embodied cognition and mathematical reasoning [6].

The focus of the current study is to investigate the ability of preschool-aged children to understand and solve fractional quantities involving wholes and one-half fractions using “equal sharing” and how that ability correlates with fine motor skills and the use of gestures. The participants included 33 preschoolers aged 4 to 5 in a Southeastern public elementary school. They were examined individually in an interview-like setting inside the school, where they completed a mathematics task developed to evaluate subjects’ ability to conceptualize, divide, and separate whole quantities into subparts. The children were presented items as pictures on a sheet of paper and were asked to split up the items “evenly” and “fairly” amongst people or other objects. The Grooved Pegboard test [7] and behavioral rating of gesture was used to assess the subjects’ fine motor ability.

### 1.1. Development of Numerical and Arithmetical Skills

The approximate number system (ANS) model suggests that we are born with the tools (intuitions regarding numbers and sets) for number processing and that through the development of new symbol systems we can build upon our foundational system of number sense [8]. Although a consensus of researchers agree on the notion of such system, it is still up for debate whether arithmetical ability arises from innate, specific cognitive skills or whether it is due to general cognitive abilities such as memory and reasoning. Authors such as Dehaene and Butterworth believe that it could be a result of our evolution, since animals such as parrots, dolphins, dogs, and monkeys can distinguish between different numerosities and even perform basic arithmetical operations. Perhaps the similarities seen between us and various animal species suggests that number approximation is a result of certain evolutionary pressures, resulting in the emergence of an internal brain system dedicated to rudimentary arithmetic.

Dehaene [8] suggests that in order for us to acquire and represent information about numerical quantities, there must be a specific cerebral circuit that facilitates this processing. Studies using lesion methods often indicate the presence of a dedicated neural system for number sense, identifying the inferior parietal region as a significant node in the circuit of the number sense system [8,9]. This network might be present in both hemispheres of the intraparietal cortex and it is thought to be involved in mental manipulation of numerical quantities, since this region displays activation while an individual performs various number processing tasks [2,10].

Lesion studies also identify the angular gyrus as a critical brain region for normal arithmetical performance; and if this area or the intraparietal region is damaged, then one would show deficits in numerical processing and arithmetic [8]. Specifically, damage to the inferior parietal region can result in impaired internal representations of quantities and an inability to comprehend the actual meaning of numbers [8]. Additionally, research conducted on individuals with developmental dyscalculia, a disorder associated with difficulty in understanding number concepts, facts, and procedures, also supports Dehaene’s argument. Evidence shows that several forms of cognitive difficulties may interfere with mathematics performance, but they are not causal features in dyscalculia. These examples indicate an innate specific capacity for acquiring arithmetical skills rather than it being a learned process throughout the course of development.

Converging evidence presented by [11] suggests that the strong association between number sense and perception and action supports the presence of a ‘sensorimotor numerosity system’ that resides in the parietal cortex. It is important to note the many similarities seen between these two systems pertaining to their proximity to each other and the large number of shared cortical regions during these two processes. This review provides examples of this interaction from neuroimaging studies, highlighting the parietal cortex to be active in both numerosity perception and action areas pertaining to eye and hand movements. We agree that in order to fully understand numerosity, it is critical to evaluate possible motor mechanisms involved in building foundational symbolic mathematical skills.

The ANS system gives us the ability to mentally represent numbers and recognize the magnitude of both symbolic (Arabic numerals, number words) and non-symbolic (unrelated dots patterns, random sticks patterns) numbers [12]. As we get older, we develop strategies to use language and symbolization to understand and work with numbers [12]. One strategy that has been extensively researched is the idea of a numerospatial “mental number line” that allows us to manipulate and calculate numbers in our head [13]. This potentially inherited system represents the idea that the difference between two consecutive numbers is the same regardless of their positions on the number line [14]. Because the mental number line helps put the weight of quantity onto an Arabic numeral, it seems to be vital for future arithmetical thinking and for calculating numbers in one’s head. With the use of language, the mental number line allows us to conceptualize numbers on a more abstract level by expanding the semantic range of the concept of number [13]. In this sense, language allows ANS representations (e.g., “five” and 5) to be mapped onto exact symbolic representations, thus reinforcing the strength between Arabic numerals and number words [2,9].

We use our number sense as a fundamental parameter by which we make sense of our world [8]. If this ability is compromised, then one could have trouble understanding and working with numbers later on. It is no surprise that having a solid foundational knowledge of mathematics is vital for future academic performance, careers, and managing day to day responsibilities such as balancing a check book. For these reasons, it is important to identify deficits early on and help educators come up with creative ways to help a child with a learning disability. However, knowing how to represent numbers does not necessarily give people the ability to solve complex, higher-order mathematics problems [12]. In regard to arithmetic, it is still unclear whether we use numerical representations to manipulate numbers themselves; some researchers suggest that the ANS is active only at the early stages of our development [12,13].

Interestingly, the study by [14] found that ANS acuity was a significant predictor of mathematics achievement at 5 years of age, but not at 7 years of age. Gimbert et al. [15] suggests that a reason for this is that as mathematical thinking advances, children begin to rely less on their ANS and more on other cognitive mechanisms. The researchers suggest that after the ANS relationship is formed, working memory assumes a larger role in mathematical reasoning [15]. Results from this study suggest that the age range between 5 and 7 may be an important transitional period where the brain seems to be switching gears from relying on ANS to working memory when working with numbers. Gimbert et al. [15] suggests that a reason for this switch from the ANS to working memory is that after we learn the magnitude of numbers, we then learn how to manipulate them while keeping in mind arithmetical rules. The differences shown between these ages suggest specific developmental processes and important changes in cognition are happening that can potentially predict an individual’s success in learning mathematics [15].

The study by Chu et al. [14] found that preschool-aged children have capabilities for both numerical abstraction and numerical reasoning. Numerical abstraction (also known as enumeration) involves a set of cognitive skills that are used to acquire representations of numerosities of sets and these skills adhere to one-to-one correspondences [14]. We use this when counting and two types of enumeration exist: subitizing and counting. Subitizing is rapid, precise, and accurate, and we use it when counting less than four items [16]. Counting is more error prone and takes longer to process, 250–350 ms per item, rather than subitizing, 40–100 ms per item [16]. Typically developing children as young as three years old have an understanding of small numerosities (up to four items) and know that counting is a way to find the total number of items in a set [9]. Enumeration is thought to give young children the tools needed to form representations of numerical quantities and discriminate different sets of numbers from each other [16].

### 1.2. Fractional Knowledge

In the initial stages of learning arithmetic, children are actively making use of their counting skills, which allows them to know that numbers have both a sequence and a numerosity (cardinal) meaning [9]. Our knowledge of fractions begins with a whole-number bias that we learn through counting and sharing [17]. Learning activities that include the number line are beneficial for teaching fraction concepts because it provides children with an integrated spatial structure to represent both whole and part number magnitudes [18,19] Theoretically, the mental number line should aid in our understanding fraction magnitude concepts because it draws on pre-existing associations between numerical magnitudes and spatial locations [19]. In addition, it also helps students see that there are an infinite number of fractions lying between whole numbers [18,19]. As a result, activities that emphasize the importance of the number line when working with part–whole relationships can make it easier for students to critically think about proportions and the arithmetical relationships between two or more fractions [19].

People use different strategies when working with fractions and a finding from Fazio et al. [18] indicates that people with difficulties concerning fraction magnitude comparison showed both a lack of conceptual understanding of correct procedures and a failure to recall correct procedures. This lack of a conceptual understanding can hinder progression in mathematics and effects can last throughout adulthood [18]. High-paying jobs in STEM exemplify this because they generally require an individual to have substantial competency in mathematics. Careers in engineering, software development, medicine, etc., all require an advanced level of proficiency in mathematics and many of the mathematical demands in these jobs are built upon fractions. These demands are omnipresent in higher-level fields of mathematics learned after fractions such as prealgebra, algebra, and trigonometry, so a solid base knowledge of these concepts is needed for success in these areas [18]. Consequentially, but not surprisingly, adults lacking a strong fractional knowledge foundation are at a loss because attempts at working with equations in these areas without understanding the fractions involved would be futile [18].

### 1.3. Executive Functioning

In the review by Cameron, Brock, Murrah, Bell, Worzalla, Grissmer, andand Morrison [20], researchers state that it is important to consider the effects of executive functioning on aspects of academic performance. Executive functioning is defined as the higher-level cognitive processes that facilitate new ways of behaving and optimizes an individual’s approach to novel situations [20]. This is essential to our understanding of mathematical cognition because executive functioning is often associated with mechanisms of working memory, in addition to problem solving, planning, and coordinating responses that require recall and organization of newly learned information in novel ways [21].

Individuals with a deficiency in mathematics tend to have problems with acquiring the basic skills needed to solve arithmetic and more contextualized assignments such as word problems, especially when these students also have problems with reading [22]. Low achievement in these areas often reflects the possible difficulties students with a learning disability in mathematics or reading have with memory and novel information processing [10]. If there were deficits in working memory, then it would be especially difficult to learn arithmetic, primarily because working memory is responsible for short-term storage of information and allows us to hold that information in our mind and work with it [23,24]. Deficits in working memory (i.e., managing, storing, and integrating more than one set of information) are common in children with various learning disorders [10].

Cognitive and metacognitive processes such as effectively processing, diagramming, and solving multistep mathematics equations are necessary when solving mathematics problems because they allow us to maintain and manipulate information while also keeping in mind the rules of arithmetic [10]. In addition, research suggests that a higher executive functioning processing capacity overall would allow someone to learn concepts and pay attention in class more efficiently [22]. This is significant especially in the classroom because behaviors that facilitate learning (following directions and sustaining attention) serve as the basis for excelling in the traditional school setting [20].

Visual representations, such as diagrams, seem to help students better understand mathematical concepts [25]. The use of diagrams has also been shown to be beneficial in managing, storing, and integrating information from math problems that require several steps [25]. Furthermore, visuospatial capacities appear to be largely active when learning a new mathematical skill and applying it later [25]. In regard to teaching mathematics, the use of visual representations can increase both mathematical understanding and processing of information, and therefore using these aides in a meaningful way can function as an instructional scaffolding strategy for students [25].

### 1.4. Fine Motor Ability

Fine motor ability entails how we coordinate precise movements of small muscles in our hands through eye coordination [20]. Without it, we would not be able to grasp a pencil, tie our shoelaces, or brush our teeth [26]. The review by Cameron et al. [20] indicates that fine motor activities play a significant role in the development of a child because they take up a substantial amount of a typical preschooler or kindergartener’s school day. For example, the researchers conducting this observational study of kindergarten classrooms found that 46% of the school day was devoted to fine motor activities. These activities included tasks such as writing, using scissors to cut paper, bean counting tasks, and playing with toys such as building blocks and Legos. Tests that are designed to evaluate fine motor ability commonly involve tasks with visual, cognitive, and manual dexterity demands (e.g., drawing a picture with a pencil) and also assesses an individual’s capability for understanding spatial organization (e.g., building with blocks) [20].

Fine motor ability is controlled by the cerebral cortex, basal ganglia, and cerebellum [26]. These areas are also increasingly being associated with mechanisms of executive functions such as working memory and general reasoning skills [25]. The cerebellum is an example of this development, being traditionally thought to coordinate motor movements but is also increasingly associated with cognitive functions such as sustaining attention [4]. For example, Diamond [4] found the cerebellum to be the most active when an individual is presented with novel, cognitive tasks. In addition, one could also credit the cerebellum with a role in numerical cognition because children eventually learn about quantity through motor-based interactions with their environment; an example of this is pointing and counting on your fingers [27].

The idea that the prefrontal cortex plays a role in both motor functions and cognitive functions is also gaining traction [4]. The prefrontal cortex is most often implicated with executive functions; however, increasing data show that this might not be its only function [4,5]. Grissmer et al. [5] suggests that it might also be involved in numerous other functions such as coordinating complex motor, emotional, or cognitive activities that requires action from several parts of the brain [5]. Functional neuroimaging studies provide evidence that when a cognitive task is being performed, activation in the dorsolateral region of the prefrontal cortex increases along with the cerebellum [4]. The primary motor cortex is also important because observations suggest that it becomes activated when solving numerical problems [4]. In short, consistent evidence that displays activation in the motor areas of the brain has shown to be a significant indicator to understanding how we solve mathematics problems [4,6]. These findings add significance to the proposition that fine motor skills and cognitive abilities are more related than previously thought and even possibly intertwined.

### 1.5. Gestures and Counting

There is a large body of evidence showing that our fingers play a major role in counting and arithmetic [28]. The act of using your fingers to count may play an essential role in learning the basics of numbers because when a child enters preschool, counting is typically taught by showing an explicit motor behavior where one is instructed to watch their fingers move while counting [29]. This association extends beyond just learning how to count because children’s arithmetical skills are best predicted by how well they perform in finger discrimination task [28,29]. This could be viewed as a cognitive advantage because the act of using finger gestures is assumed to ground thought in action by reducing demands on working memory [30]. Perhaps this reduction in cognitive workload allows the brain to multitask more efficiently, thus assisting mechanisms necessary for paying attention and participating in classroom instruction.

Numerous behavioral and neuroimaging studies have confirmed that mental arithmetic relies on pre-existing representations in the sensorimotor system. However, many questions remain. One association still open to interpretation regards the possible overlap between brain areas involved in mental arithmetic and those involved in finger discrimination. Many researchers agree that perhaps fingers constitute a useful means for obtaining and communicating arithmetic knowledge because they offer a physical counterpart for mental operations. Whichever the case, it is widely agreed that these fine motor systems give us the essential motor skills to solve mathematics problems in school and these associations typically extend into adulthood.

Gesturing during mathematics instruction can help students better retain concepts because it can contribute representational, supplemental information that may not be conveyed by verbal language alone [20,31]. Interestingly, a growing amount of research suggests that mathematical processes are supported by the sensorimotor system [29]. Future examination of the neural systems involved when an individual solves mathematical problems is necessary because more data are needed to prove that a dedicated sensorimotor circuit is in place and is active when we work with mathematical concepts [29].

The studies by Susan Goldin-Meadow imply that the use of hand gestures during math instruction can facilitate learning [30,31]. These studies indicate that the use of gestures can reflect an individual’s cognitive state and perhaps can play a causal role in learning mathematics [25,32]. In order to distinguish this from a correlational to a causal relationship, the use of gesture can be manipulated in studies when instructing children to use or restrict gestures while solving math problems [25]. Perhaps gesturing plays a role in memory and learning in both children and adults because they can aid in retaining knowledge in a way that decreases demands on working memory, freeing up cognitive resources that can be used elsewhere [31]. This finding is especially beneficial to children with a learning disability in mathematics because representational gestures have been associated with learning when solving math problems. These children often struggle with working memory, so encouraging the use of gestures during math instruction may be a helpful intervention to decrease cognitive load and facilitate learning by helping children extract information from their own hand movements during counting tasks [31].

### 1.6. Fine Motor Skills and Cognitive Development

Previous literature has also shown that fine motor ability is a strong predictor of academic performance [4,5,24]. Cameron et al. [20] suggest that if a child has a deficit in fine motor development, often times their overall academic performance suffers as well. As a result, those children are more likely to take longer to complete assignments and fall behind in school [20]. Additionally, most activities that build or demonstrate cognitive skills also involve fine motor tasks (e.g., reading and writing) [5]. Fine motor ability is implicated in reading because it requires finely tuned psychomotor control necessary for eye tracking and directing one’s eye movements during word tracking [5]. In like manner, fine motor control over the hands as well is necessary for hand–eye coordination when writing [5]. Concurrent research also implies that cognitive and motor functions display equally protracted time courses during development [4]. Evidence for this comes from studies such as Diamond [4], which demonstrate that both motor and cognitive systems are affected when there are genetic or environmental perturbations.

One reason why studying preschoolers is important to our understanding of cognitive development is because when a child is young, they are most amendable to learning [20]. This time point is often called the “sensitive period” and can be described as the optimum window of opportunity for brain malleability and is thought to occur just before or around the age of seven years old [26]. This part of a child’s life is when they are the most receptive to cognitive development and these changes often extend into adolescence and adulthood [26]. During this sensitive period, the method in which an individual learns mathematics can drastically alter future mathematics performance due to the fact that they are actively developing the foundation for mathematical reasoning and are beginning to scaffold fractional understanding concepts [27]. In addition, researchers who have explored the long-term effects of early interventions have concluded that the early years are the most cost-effective time to intervene [20]. Moreover, identifying and fixing these kinds of deficits in a child’s development during these years could provide a strong base for future mathematics reasoning abilities.

### 1.7. Fair Sharing

A critical cognitive milestone during the preschool years is the concept of fairness [3]. This hallmark of social cooperation is defined as the ability to share items in a way that agrees with standard principles of justice [3]. Children this age show a dilemma when it comes to fair sharing because although they understand codes of fairness by 3 years of age, they do not always exhibit these behaviors [33]. This “moral hypocrisy” known as the knowledge–behavior gap, assumes that children know they should share resources fairly but are often reluctant to when sharing deals with the first person [33]. Chernyak et al. [3] suggest that this is a cognitive process that is poorly understood, but perhaps it can be explained by the insufficient cognitive resources hypothesis. They state that although children generally understand number cardinality (the number of items in a set) at this age, they might be missing the cognitive abilities that enable them to behave in accordance with social expectancies [3]. These resources may still be in early development, and tasks such as resource distribution require the coordination of advanced behavioral and cognitive abilities that may not be fully established yet [3].

Furthermore, results from [3] suggest that numerical cognition plays a role in sharing behaviors. For example, in order to state that a distribution is fair, the child must have numerical cognition systems in place that allow them to understand the rule of cardinality [3]. If the child distributes two batteries to one flashlight and two batteries to another flashlight, then that distribution would be fair because it shows cardinal equivalence [3]. This may help explain the cognitive mechanisms at work when conceptualizing ideas such as fairness and equality.

Cwikla [34] was interested in young children’s naïve understanding of fair sharing and the differences in strategies used for solving contextual problems before they had any formal instruction in school. Children this age are likely sharing and partitioning snacks and toys amongst their siblings and peer groups, so she also questioned whether these problems are best presented in the context of fair sharing. Upon reviewing the literature, Cwikla found that minimal empirical evidence exists as to whether three, four, and five-year-old children can understand or acquire such fractional concepts before formal instruction in school where the whole-number bias is established. Previous research [9] suggests that children have some knowledge about partitioning objects when they first enter school, adding to the claim that our number sense is somewhat innate. Cwikla tested children aged three to six on how well they described and illustrated their attempts at fair sharing tasks. She would read a question such as “Chris wanted to share six crackers with his three friends. How could he do this fairly?” and prompt the child to draw the items and show how they would share them fairly. All of these questions were framed socially, using the snack-sharing context as something the child would find familiar. Student work from this study shows that prekindergarten students have the ability to consider, illustrate, and explain fair-sharing tasks with mixed fraction solutions. Although children at this age may not use the proper mathematical language to express their solution to these problems, they still more often than not understand when something has been fairly distributed. These results suggest that preschool-aged children have an intuitive understanding of fractional quantity and understand the notions of “fairness” and “sharing” before formal mathematics instruction.

### 1.8. Current Study

The purpose of the current study is to use these findings to extend the work of Cwikla [34] by examining whether general developmental factors of age and fine motor ability predict the development of fractional reasoning and fair sharing concepts in preschool-aged children. Because a child’s implicit knowledge of simple arithmetic is supported by the approximate number system, it is important to identify the cognitive mechanisms that facilitate the development of numerical reasoning [15]. The ability to consider the parts and the whole simultaneously appear around age six or seven, although evidence [34] suggests that the age of acquisition may be earlier. In addition, we investigated whether children who show signs of weak fine motor skills also have lower scores on our measure of cognitive abilities such as fractional reasoning and subitizing. Understanding the precursors of fraction understanding and the cognitive load that goes into solving problems involving fractions will allow researchers to gain more insight into this relationship which can potentially help teach educators the most effective and evidence-based ways of promoting a solid foundation for numerical reasoning.

The current study proposes that the development of mathematical competence, specifically fractional reasoning, is a multifaceted process that requires integration from executive functioning skills, fine motor ability, and the use of gestures. Indeed, if the neural substrates for enumeration are overlapping with those of fine motor control, as suggested by Soylu, then one would expect a significant correlation between these two behaviours to develop early on when basic counting is so critical for math task performance. The ability to control and direct the operations of the hand and fingers may result in better counting performance but may not directly influence mathematical cognition. This study examines video recordings of the gestures made during a dot counting task to yield an objective measure of fine motor ability that goes beyond that of motoric speed, and looks for signs of overall fine motor development as well as motor/cognitive synchrony. This is assessed through ratings of the child’s hand morphology, accuracy of gestures, and synchrony of both gestures and spoken word.

### 1.9. Hypotheses

In terms of specific hypotheses, we predict a (1) similar result from Cwikla [34] in that preschool-aged children will successfully conceptualize and solve simple fractions. We also predict that (2) a positive correlation between fine motor ability and performance on the mathematics task will emerge. Finally, we (3) predict that gestural ability will correlate with scores on the mathematics task and with performance on the Grooved Pegboard Task

## 2. Materials and Methods

### 2.1. Participants

Participants were 33 children aged four to five in two preschool classrooms in a public school (age range 4 years, 0 months to 5 years, 11 months; mean = 4.66 years). The school is located in a town in Southeastern Alabama and the residents have an average median household income of $40,020 annually. The population is predominately African-American, accounting for 50.6% of the population. Forty-five percent of the population is White, 1.8% is Asian, 1.4% is mixed, and 2.4% are of Hispanic or Latino origin. Native Americans and other races account for less than 1% of the total population (U.S. Census data). The preschool classes consisted of approximately 15–19 students each, both classes were taught by an experienced teacher and a teacher’s aide.

### 2.2. Materials

#### 2.2.1. Mathematics Sharing Stories

**Figure 1:**
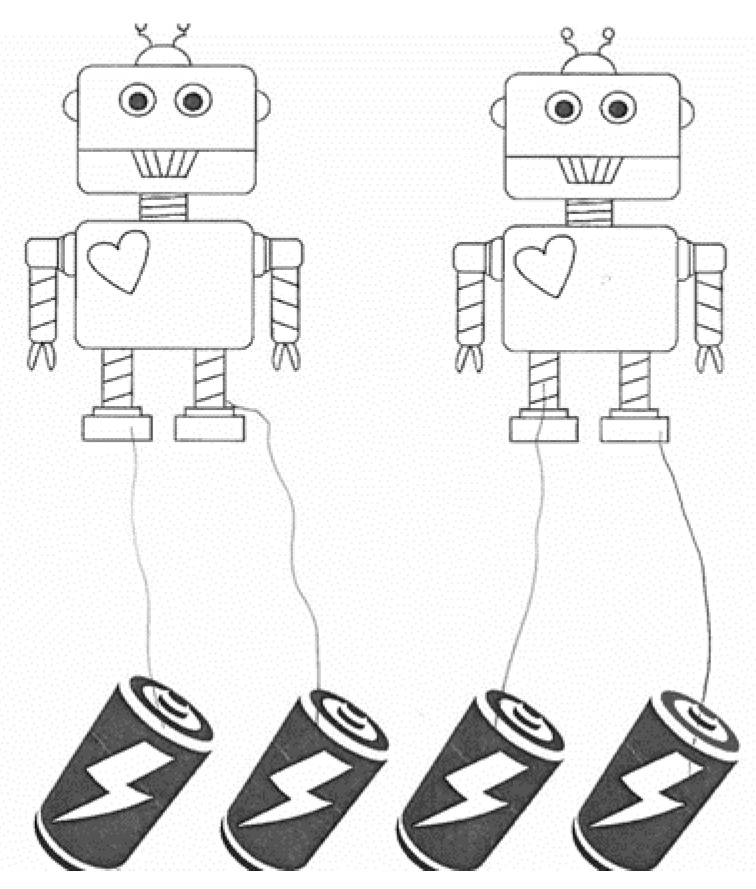
Example of a correct mathematics story response. Note. Student work showing a correct distribution of four batteries to two robots. This response received a score of 8, meaning that all of the batteries were distributed correctly and fairly, and no partitions were necessary.

**Figure 2:**
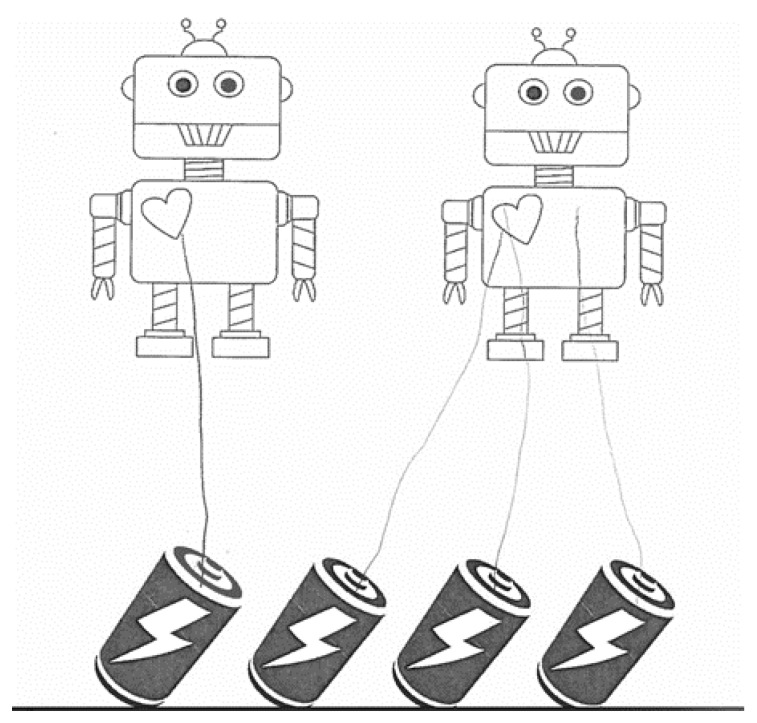
Example of an incorrect mathematics story response. Note. Student work showing an incorrect distribution of four batteries to two robots. This response received a score of 5, meaning that the batteries were all distributed correctly as wholes, but not fairly.

The mathematics sharing stories were developed similar to Cwikla’s [34] study, where the child was asked to show or illustrate how to share various items with other objects fairly. There were nine questions in total. The experimenter read each question to the child and then asked them to draw on the paper showing how they would share the items. Questions were similar to the following: “Here we have two robots and four batteries. Show me how you would share all of the batteries so that each robot gets the same amount.” The child would then draw a line connecting the batteries to the robots. An example of a correct response is shown in Figure 1 and an incorrect response is shown in Figure 2. The child’s response shown in Figure 1 would receive a score of 8, meaning that all of the batteries were distributed correctly and fairly, and no partitions were necessary. The child’s response shown in Figure 2 would receive a score of 5, as s/he split up wholes correctly, but not fairly. The question (distribute four batteries to two robots) only regarded wholes; therefore, an 8 is the highest score one could make on this question. A 9 or 10 would have not been achieved because no extra partitions were necessary, unlike other items that involve partitioning objects and splitting the parts amongst people or other objects (See Figure 1 and Figure 2).

After the child answered the question, the experimenter would ask whether their distribution was “fair” and “equal”. The children completed one trial, consisting of nine questions. Out of the nine questions, two of them (Question #5 and Question #9) required a partition and a distribution; the other seven questions dealt with whole numbers only (See Appendix A). Other than being asked to draw a line on the paper to indicate how they would distribute the items, there were no other instructions given to the subjects. The experimenter also made no corrections to the child’s work and minimal feedback was given.

#### 2.2.2. Dot Counting Task

Next, a dot counting task created by the researchers was administered. The children were presented a piece of paper consisting of four boxes, each consisting of a various array of dots. There were four cards and 16 total dot arrays. The child was instructed to count the number dots out loud while using their finger to make gestural points. The number of dots in the 16 boxes ranged from 2 to 9 (See Appendix B). The children were rated between one and three on whether they initially subitized, in addition to their hand morphology, accuracy of gestures, and synchrony of both gestures and spoken word.

#### 2.2.3. Grooved Pegboard Test

The Grooved Pegboard test [7] measured both motor speed and hand–eye coordination. It is a widely used metric that requires refined manual dexterity for completion. The pegboard apparatus is made up of a metal surface with a 5 by 5 matrix of keyhole-shaped holes in various orientations. The pegs have ridges on the sides that need to be twisted around or oriented differently in order to fit the pegs into the grooved holes. During the task, the child is told to insert all of the pegs into the 25 holes on the pegboard. They were also told to go as quickly as they can, use only their right or left hand, start from left to right, and pick up one peg at a time. The subjects completed two trials, first with their dominant hand and second with their non-dominant hand. Specific hand preference was evaluated by which ever hand the child used to draw during the mathematics story task. The score is calculated from the amount of time taken to complete all of the rows and also the number of pegs dropped.

### 2.3. Procedure

In order to test the hypotheses, we used a panel design, where all students received the same stimulus. Hypothesis 1 was descriptive, 2 and 3 were correlational. In order to test the descriptive and correlational hypotheses in this study, a pre-experimental single sample design was used in which each child was assessed one time, individually. All students in the classroom were afforded opportunity to participate, and no experimental variable was manipulated. An IRB was obtained from the University of Southern Mississippi and the study is funded by a $902,000 grant from the W.K. Kellogg Foundation of Battle Creek Michigan (No. P3035020) titled “Interdisciplinary Mathematics and Literacy e-Stories for Young Learners Mathematics sharing stories”. Children were examined individually in an interview-like setting at school, presented with items as pictures on paper and tasked with portioning items evenly and fairly amongst people or objects. This task was the most time-consuming measure in this study, taking on average 45 min for the students to finish. For each of the nine math problems, children were evaluated on how well they solved the mathematics task and scored between 0 (no defined strategy) and either 8 (distribute all wholes correctly) or 10 (distribute wholes and partitions correctly) for each task. Questions #5 and #9 were the only problems suitable to score a 10 because they require the child to make a partition and distribute, unlike the other questions that require only a whole-item distribution. Children’s responses were coded and analyzed in terms of framing the problem, identifying the parties involved, using fractional reasoning (parts), performing segmentation, and accurate sharing. The current experiment lacks a control group because the aim of this study was descriptive and correlational, first seeking to establish the ability of 4-year-old children to solve fractional reasoning problems and also to establish whether this ability is correlated with motor skills.

Following the mathematics story questions was a dot counting task. A “warm up” trial consisted of the experimenter asking the child to count to ten on their fingers as fast as they could. After, the child was presented a card with four boxes containing black dots. They were instructed to use their finger to count the number of dots in the box while also counting out loud. This task was relatively short in duration, taking approximately two to five minutes to accomplish. The experimenter took a behavioral rating of gesture looking at hand morphology, accuracy of gestures, and synchrony of the child’s verbal count and number of gestures made. The experimenter also recorded whether the child subitized (looked at the dots and knew how many there were without explicit counting behaviors) but the child was not instructed to count this way. The child scored between a 0 and 3 for each of the gesture variables. Two research assistants analyzed videos of the child performing the task in order to determine the reliability of coding. Their coding correlated between 0.92 and 0.97 for the four observed variables (subitizing, hand morphology, accuracy, and synchrony).

The final task the subjects completed was the Grooved Pegboard test, which evaluated their fine motor ability. The time taken by the child to fully complete the pegboard test and the number of times a peg was dropped by the child were recorded for both dominant and non-dominant hand trials. Students typically took between two and four minutes for each trial and the experimenter capped the time allotted per trial at six minutes.

Data were entered into Google Sheets for initial editing and storage using a secure, password-protected file with no unique identifiers. Participant identifiers were kept in separate file only shared with key personnel. The statistical program “SPSS” was used for analysis. 

## 3. Results

### 3.1. Hypothesis 1

In order to test Hypothesis 1, which was that children have the ability to conceptualize and solve simple fractions, we performed a descriptive analysis on the mathematics stories total scores that produced a maximum score of 76 and a minimum score of 8 (See Table 1). The mean mathematics story score was 40.36 points out of 76, indicating that participants scored 53.11 percent correct on average (See Table 1).

In order to determine whether the children performed better than chance, a priori baseline probability of mathematics total score was calculated. Each of the nine items had different probabilities of achieving a correct answer if the children were simply guessing. For instance, there were three keys which represent the a priori A category and three keys which represent the a priori B category. In this example, the multiplicative combination of both a priori events getting the correct answer was 10.89 percent (See Table 2). The a priori baseline probability of math stories total score was computed to be 12.46 out of 76 (See Table 2).

A scoring rubric was created to assess children’s accuracy on the mathematics sharing stories (See Table 3). In order to achieve an appropriate level of interrater reliability, three university students were recruited to score the mathematics stories by reviewing video recordings of the child performing the task and following the scoring rubric protocol.

In order to test the hypothesis that children would be able to conceptualize and solve simple fractional reasoning problems, a one-sample *t*-test was conducted using the total score as the dependent variable and the a priori accuracy rate of 12.46 as the comparator. The Shapiro–Wilk test for normality indicated no violation of the normality assumption, and thus the parametric *t*-test was employed, W (33) = 0.975, *p* = 0.200. This analysis was significant, supporting the hypotheses that children performed a rate significantly greater than chance on the complex story problems, t(32) = 9.771, *p* < 0.001 (See Table 4).

In conclusion, data support Hypothesis 1 in that children performed better than chance on our mathematics measure, showing some ability to solve and conceptualize problems involving whole numbers and fractions.

### 3.2. Hypothesis 2

To examine Hypothesis 2, correlations were conducted to examine the relationship between fine motor ability and performance on the mathematics task (Due to time constraints, the experimenter implemented a cap at 6 minutes (900 s) taken to complete each trial. Four children did not complete the trials. For data analysis, their results were interpolated based on the number of rows on the pegboard completed in 900 s (See Table 5)). Math total score correlated significantly with pegboard dominant hand time, non-dominant hand time, and non-dominant hand drops (See Table 6).

Descriptive analyses found the mean time taken to complete this task with their dominant hand was 243.18 and 271.82 s for their non-dominant hand (See Table 6). Number of peg drops during the task was between one and ten for both dominant hand and non-dominant hand trials (See Table 6).

A linear regression was conducted to display that fine motor ability, as measured by the Grooved Pegboard test, predicts scores on our mathematics measure (See Table 7). The outcome variable was total score on the mathematics stories and the independent variables were pegboard dominant hand time, non-dominant hand time, dominant hand drops, and non-dominant hand drops. As visible in Table 7, the standardized beta value was −0.508, with the *t*-test for step 1 of the regression being −3.281, and a significant p value of 0.003. the non-dominant drops yielded an adjusted R-squared value of 0.234, indicating that the number of non-dominant hand peg drops explains 25.8% of the variance of the dependent variable (mathematics story total score). R-squared change(1,31) = 0.258, *p* = 0.003. When excluding non-dominant drops, the linear regression found no other pegboard variables to be significantly correlated with mathematics performance (See Table 7). Data analyses support Hypothesis 2, showing a positive relationship between fine motor ability and performance on the mathematics task.

In order to account for the effect of developmental age on our primary variable of interest (mathematical story total score), the child’s age at the time of test was entered into an additional multiple linear regression as a predictor along with all pegboard variables, as above. Non-dominant drops emerged as the sole, significant predictor in model step 1, R2adj = 0.292, F change (1,26) = 12.151, *p* = 0.002, and an adjusted beta of −0.564. Age at time of test did emerge in step two of the model with an increased R^2^adj of 0.417, indicating that the addition of age accounted for a significant 12.5% more of the variance in mathematics total score, R^2^change = 0.142, Fchange (1,25) = 6.55, *p* = 0.017, and standardized beta for Step 2 age = 0.384.

### 3.3. Hypothesis 3

To test Hypothesis 3, which was that gestural ability would predict scores on the mathematics task and correlate with performance on the Grooved Pegboard test, children’s performance was scored between 1 and 3 on subitizing, hand morphology, accuracy, and synchrony for each of the 16 dot arrays (See Table 8).

Descriptive analyses produced mean scores of 1.25 for subitizing, 2.57 for hand morphology, 2.23 for accuracy, and 2.34 for synchrony (See Table 9). Analysis for subitizing, hand morphology, accuracy and synchrony produced minimum, maximum, standard deviation, skewness, and kurtosis values are detailed in Table 9.

Due to the ordinal nature of the gestural ability variables, Spearman’s Rho correlations were used to measure the strength of the association between these variables and measures of performance on the Grooved Pegboard test. Tests of normality were conducted to check for any violations of normality within the gesture variables. Z-scores for the gestural ability measures were calculated because an analysis of the data determined a significant level of kurtosis and skewness for the above variables (See Table 10).

Z-scores for subitizing, accuracy, hand morphology, and synchrony were correlated with performance on the mathematics task (See Table 10). Math story total score correlated significantly with subitizing (*p* = 0.022) and accuracy (*p* = 0.015), but not hand morphology (*p* = 0.609) or synchrony (*p* = 0.169) (See Table 11). Accuracy was the most significant correlate of the Grooved Pegboard test, with *p*-values of 0.002 for both dominant hand time and non-dominant hand time but produced insignificant values for dominant hand drops (*p* = 0.597) and non-dominant hand drops (*p* = 0.090). Synchrony only correlated with dominant hand time (*p* = 0.006) (see Table 11. Hand morphology and subitizing produced no significant *p*-values for all Grooved Pegboard variables.

Hypothesis 3 was partially supported by the data, showing that children’s gestural ability (subitizing and accuracy only) predicts performance on the mathematics task. Five children did not complete this task and were excluded from the data set.

## 4. Discussion

Our goals in this study were to gain insight into preschool-aged children’s capability of understanding and manipulating fractional quantities and to determine whether fine motor coordination and the use of gestures is correlated with our measure of mathematical ability in preschool aged children—specifically within the context of understanding fair sharing. The general results are in accordance with a common finding in the literature that cognitive and motor skills progress at a similar rate in early childhood development. While the direction of causality has not been established, our finding of significant correlations between these constructs supports the notion that intervention in one domain may support development of the other.

Hypothesis 1 asked whether pre-K 4- and 5-year-old children would show evidence that they could comprehend fair sharing rules and basic fractional reasoning. Our analyses supported Hypothesis 1, such that children indeed demonstrated the capacity to solve fraction concepts, particularly within the context of sharing and division of items amongst people or items. This is a similar finding to Cwikla [34], implying that the age of fractional knowledge acquisition may be earlier than what is traditionally agreed upon by the educational community. Children in the U.S. are typically taught fractions around the third grade, so this discovery is noteworthy because teaching these concepts earlier may potentially help reinforce our fractional knowledge so that it extends into higher-level areas of mathematics such as algebra.

Correlations and a linear regression supported Hypothesis 2, indicating that fine motor ability, as expressed by Grooved Pegboard dominant hand time, non-dominant hand time, and number of pegs dropped by the non-dominant hand significantly correlated with our measure of fractional ability. These data are in agreement with findings from Cameron et al. [20], which indicated that a child’s level of fine motor ability correlated with academic performance. This proposes an important developmental role for tasks that promote fine motor development in mathematics instruction during the preschool and kindergarten years. The fact that age predicted mathematics total score was not surprising, and in fact reassuring in terms of supporting the validity of our new measure. Most importantly, motor ability was still a significant predictor of mathematics ability over and above age in our regression, suggesting that there is a unique relationship between the two, and that knowing a student’s level of fine motor development is better predictor of mathematics ability, as we define it here, than simply age alone.

For Hypothesis 3, a behavioral rating of gesture looking at subitizing, hand morphology, accuracy, and synchrony during our counting and gesturing task provided insight into how the use of gesture aids with mathematics learning. A correlational analysis was conducted to assess the magnitude of the relationship between gestures, fine motor ability, and mathematics performance in our sample of children. Our third hypothesis was partially supported, indicating that our gesture variables (accuracy and synchrony only) correlated significantly with Grooved Pegboard test variables. Subitizing and accuracy were the only gesture variables that significantly correlated with mathematics stories total score.

Results from Hypothesis 3 add to recent findings signifying that integrating hand gestures during mathematics instruction may promote learning by playing a supporting role in counting and subitizing. Data from the current study signify a relationship between accuracy of hand gestures during a counting task and performance on our mathematics measure. This is instructive because encouraging the use of hand gestures when counting could potentially offer educators a technique for improving learning in their students.

Recent findings show that cognitive and motor functions display equally protracted time courses during development, which suggests that this relationship may persist throughout childhood [4]. It is also believed that motor skill development could have a significant impact on cognitive development [27]. Together, these results insinuate that a child’s dynamic interaction with their environment across development is important for learning [35].

Research on the neural and cognitive mechanisms that provide us with the tools necessary to conceptualize numbers is instructive to education research because an insufficient foundational knowledge of numerosities (place value, addition, and subtraction) may lead to difficulties with mathematics in later years [19]. Individuals lacking a solid footing in mathematics often struggle with connecting essential, introductory arithmetical rules to more intricate problem-solving methods that is required as they progress through mathematics instruction [19]. Consequentially, they are at a considerable disadvantage when it comes to more advanced schoolwork and succeeding in future careers. Interventions have been shown to work so additional investigation into this relationship should encourage the future development of training methods to help those with deficiencies in mathematics. Results from this study may also help with educating teachers on the most effective ways to teach children mathematics. This knowledge broadens our understanding of what are the most effective and evidence-based ways of promoting a solid foundation for numerical reasoning in children.

Discoveries from the current study and others previously cited help us gain a better understanding of the higher-level cognitive mechanisms that are required to understand rules of numbers and arithmetic and eventually to solve fraction problems. For example, the development of cognitive systems such as working memory is necessary when working with numbers because it gives us the ability to store and process material over short periods of time [36]. It is also conceivable that skilled counting practices require simultaneous use of multiple components of working memory [19]. Because working memory assumes a greater responsibility in learning around age four, it is important to consider its role in significant areas of learning for children such as reading and mathematics [36]. In like manner, the emergence of these cognitive systems is often credited with helping humans evolve necessary social and functional adaptations to one’s environment [37]. Several studies point to a possible overlap of these results with the phenomenon of “groupitizing,” [38,39], the phenomenon that participants are faster and more accurate when enumerating an array of items when these can be grouped into subgroups according to some rules (e.g., spatial or temporal proximity, and color). This phenomenon is thought to be related to the ability to divide the whole into subitizable subgroups and to mental calculation abilities. Starkey and McCandliss [38] found no evidence of grouping in their kindergarten age sample, whereas we see some evidence of it in recordings of gesture in our 4–5 year olds, suggesting the possibility that groupitizing may emerge earlier than thus far reported in the literature.

## 5. Conclusions

In conclusion, these findings are relevant in their support and extension of previous research that suggests that when fine motor development is perturbed, cognitive development is often affected too [14]. Outcomes of this study also provide insight into the strength of the relationship between fine motor development and cognitive development and how mathematics tasks can serve as an early indicator. These data also strengthen research at the intersection of the fields of childhood education research, cognitive science, and developmental psychology. Implications of these results also apply to clinical psychologists as well as early childhood educators. Elementary teacher mathematics training and development should build upon these noteworthy findings linking fine motor skills and mathematical sense making, considering the cumulative nature of mathematics. In conclusion, future development of a standardized and structured fractional reasoning and fair-sharing protocols should be pursued as a result of this study. Limitations of this study include not examining differences between gender, children’s age in months, and left versus right handedness. The current study employed a single testing epoch and non-experimental correlations to assess the relationship between fine motor ability, mathematics performance, and gestures in preschool children. We hope to resume testing in the schools and collect follow-up data. The focus of the current study was to determine associations between the three critical measures; therefore, we cannot determine the direction of causation.

There are many future directions we are planning on taking with this study. Research on an “e-story” format of the same questions used in this study is currently underway. Instead of the paper and pencil format, children will use a touchscreen tablet or laptop to solve these problems and will be scored on time taken to complete the task and how many attempts it took to achieve the correct answer. Preliminary data analyses of this task compared to the paper and pencil format indicate that the E-stories yield significantly better accuracy than the paper and pencil version, suggesting that embedding fractional concepts within a social context and “e-story” format may be more beneficial to mathematics learning during these early, malleable years. Another direction for this study is to produce physical manipulatives of the sharing items. For this protocol, the children will actually cut a piece of string with scissors and distribute four batteries to two robots. Children’s ability to solve this task will then be compared to performance on the paper and pencil and e-story formats. This analysis will give a better understanding of which method is the most effective when teaching children fractions.

## Figures and Tables

**Table 1:** Math Sharing Stories Descriptive Statistics.

Measure	Minimum	Maximum	Mean	Std. Error of Mean
Math Stories Total Score	8	76	40.36	2.85

**Table 2:** Computing Baseline Probability.

Task	A Priori A	A Priori B	A × B		
Locks	0.33	0.33	0.1089		
Robots	0.25	0.5	0.125		
Go Karts	0.1667	0.5	0.08335		
Cut Rope	1	0.5	0.5		
Tents	0.2	0.5	0.1		
Hats	0.5	0.5	0.25		
Oars	0.25	0.5	0.125		
Flashlights	0.1667	0.5	0.08335		
Granola	0.2	0.5	0.1		
			**Total A × B**	**Total Items**	**Combined Accuracy**
			0.1639555556	76	12.46062222

**Table 3:** Math Stories Scoring Rubric.

Score	Rubric
0	some esoteric, not previously defined strategy, such as drawing lots of lines randomly
1	add or subtract friends/items, but fail to create a fair distribution/perfect correspondence
2	draw lines incorrectly without partitioning (ex. One tent has four lines drawn from it to four pieces of cloth, but the other tent has only two lines drawn from it to two pieces of cloth)
3	add friends/items such that there is a perfect correspondence/fair distribution of items
4	draw lines correctly from items to friends/containers, but without partitioning (ex. Draw three lines from one robot to three batteries and then draw three lines from a second robot to three batteries)
5	distribute all wholes or pieces but not fairly
6	distribute wholes or pieces fairly but leave some out
7	distribute wholes then partition incorrectly or distribute pieces incorrectly
8	partition all correctly
9	distribute wholes/extra partitions
10	distribute wholes/partition economically

**Table 4:** T-Test for Math Stories.

One-Sample Test Test Value = 12.46	t	df	Sig. (2-Tailed)	Mean Difference	95% Confidence Interval of the Difference	
					Lower	Upper
**Math Total Score**	9.771	32	0.000	27.90364	22.0868	33.7205

**Table 5:** Grooved Pegboard Descriptive Statistics (n = 33).

Title	Minimum	Maximum	Mean	Std. Deviation
Dom Time	106	900	243.18	164.382
Dom Drops	1	10	4.03	2.555
Non Dom Time	121	900	271.82	172.396
Non Dom Drops	1	10	4.55	2.538

**Table 6:** Math Stories and Grooved Pegboard Correlations (n = 33).

Measure	Pearson r	MathStories Total Score	Dom Time	NonDom Time	Dom Drops	NonDom Drops
Math Total Score	r	1	−0.373 * 0.032	−0.386 * 0.027	−0.324 0.066	−0.508 ** 0.003
Dom Time			1	0.937 **	0.626 **	0.543 **
				0.000	0.000	0.001
Non Dom Time	r			1	0.665 **	0.624 **
					0.000	0.000
Dom Drops	r				1	0.628 **
						0.000
Non Dom Drops	r					1

* Correlation is significant at the 0.05 level (2-tailed); ** Correlation is significant at the 0.01 level (2-tailed).

**Table 7:** Linear Regression for Math Stories and Grooved Pegboard variables.

Step 1	Beta	t	Sig.						
Constant		10.66	0.000						
NonDomDrops	−0.508	−3.28	0.003						
**Step 2**	**R Square**	**Adjusted R Square**	**Std. Error of the Estimate**	**R Square Change**	**F Change**	**df1**	**df2**	**Sig. F Change**	**Durbin-Watson**
0.508 a	0.258	0.234	14.35909	0.258	10.767	1	31	0.003	2.155
**Step 3**	**Beta In**	**t**	**Sig.**	**Partial** **Correlation**					
Dom Time	−0.138 b	−0.745	0.462	−0.135					
Dom Drops	−0.008 b	−0.038	0.970	−0.007					
NonDom Time	−0.113 b	−0.562	0.578	−0.102					

a. Predictors: (Constant), NonDomDrops. b. Dependent Variable: Math Total Score.

**Table 8:** Gesture Scoring System.

Subitize	Accuracy	Morphology	Synchrony
	0-no strategy/makes random gestures	0-No hand used to count	0-No strategy
1-Did not subitize	1-Less than half correct	1-Limp, floppy hand, poor muscle tone	1-Says correct number/none or wrong gestures used
2-Subitized incorrectly	2-More than half correct	2-Partially tucked fingers, no single pointing digit	2-Correct gestures/incorrect number spoken
3-Subitized correctly	3-Correct number of gestures made	3-Formed hand, tucked fingers, single pointing digit	3-Correct gestures and correct number spoken

**Table 9:** Gestural Ability Descriptive Statistics.

Title	Min	Max	Mean	Std. Error of Mean	Std. Deviation	Skewness	Kurtosis
Subitize	1.0000	1.9375	1.2455	0.05133	0.27159	1.190	0.761
Accuracy	0.2500	2.9375	2.2254	0.15667	0.82904	−1.259	0.285
Morphology	0.3750	3.0000	2.5691	0.13873	0.73408	−1.824	2.357
Synchrony	0.4375	3.0000	2.3437	0.15465	0.81836	−1.229	0.160

**Table 10:** Correlations between Gestural Ability and Grooved Pegboard Performance (n = 28).

Variable	Subitize Z	Accuracy Z	Morphology Z	Subitize Z
Dom Time	−0.045	−0.554 **	−0.266	−0.190
Sig (2-tailed)	0.820	0.002	0.170	0.333
Dom Drops	−0.010	0.104	0.145	0.122
Sig (2-tailed)	123	123	123	123
NonDom Time	−0.132	−0.567 **	−0.126	−0.366
Sig (2-tailed)	0.502	0.002	0.524	0.055
NonDom Drops	−0.244	−0.326	−0.049	−0.170
Sig (2-tailed)	0.210	0.090	0.805	0.388

** Correlation is significant at the 0.01 level (2-tailed).

**Table 11:** Correlations between Math Story Total Score and Gestural Ability Measures (n = 28).

Title	Title	Math Total Score	Subitize Z	Accuracy Z	Morphol Z	Synchrony Z
MathTotal Score	Correlation Coefficient	1	0.431 *	0.455 *	0.101	0.268
	Sig (2-tailed)		0.022	0.015	0.609	0.169
Subitize Z	Correlation Coefficient		1	−0.006	−0.083	−0.147
	Sig (2-tailed)			0.977	0.674	0.454
Accuracy Z	Correlation Coefficient			1	0.619 **	0.799 **
	Sig (2-tailed)				0.000	0.000
Morphol Z	Correlation Coefficient				1	0.734 **
	Sig (2-tailed)					0.000
Synchrony Z	Correlation Coefficient					1
	Sig (2-tailed)					

* Correlation is significant at the 0.05 level (2-tailed); ** Correlation is significant at the 0.01 level (2-tailed).

## Data Availability

Not applicable.

## Appendix A

Question 1. 3 keys and 3 locks

Question 2. 2 robots and 4 batteries

Question 3. 2 go-karts and 6 wheels

Question 4. Cut rope evenly and distribute

Question 5. 2 tents and 5 pieces of cloth

Question 6. 2 kids and 2 hats

Question 7. 2 kids and 4 oars

Question 8. 2 flashlights and 6 batteries

Question 9. 2 kids and 5 energy bars

## Appendix B

Card 1.

Card 2.

Card 3.

Card 4.
